# How to report and make sense of a new HIV-1 circulating recombinant form?

**DOI:** 10.3389/fmicb.2024.1343143

**Published:** 2024-02-21

**Authors:** Zhenzhou Wan, Chiyu Zhang

**Affiliations:** ^1^Medical Laboratory of Taizhou Fourth People’s Hospital, Taizhou, China; ^2^Shanghai Public Health Clinical Center, Fudan University, Shanghai, China

**Keywords:** HIV-1, circulating recombinant form, unique recombinant form, prevalence, origin, subtype, recombination breakpoint, genomic sequence

## Abstract

Co-circulation of multiple HIV-1 subtypes in the same high-risk groups leads to the on-going generation of various inter-subtype recombinants, including unique (URFs) and circulating (CRFs) recombinant forms, which brings a new challenge for the prevention and eradication of HIV/AIDS. Identification and prompt reporting of new CRFs will provide not only new insights into the understanding of genetic diversity and evolution of HIV-1, but also an early warning of potential prevalence of these variants. Currently, 140 HIV-1 CRFs have been described; however, their prevalence and clinical importance are less concerned. Apart from the mosaic genomic maps, less other valuable information, including the clinical and demographic data, genomic sequence characteristics, origin and evolutionary dynamics, as well as representative genomic fragments for determining the variants, are available for most of these CRFs. Accompanied with the growing increase of HIV-1 full-length genomic sequences, more and more CRFs will be identified in the near future due to the high recombination potential of HIV-1. Here, we discuss the prevalence and clinical importance of various HIV-1 CRFs and propose how to report and make sense of a new HIV-1 CRF.

## 1 Introduction

HIV/AIDS is still a major global threat to human health. According to the UNAIDS Global HIV & AIDS Statistics Fact Sheet,^1^ 39 million (33.1–45.7 million) people were living with HIV worldwide in 2022, and they are infected by various HIV-1 genotypes and recombinants (Hemelaar et al., 2019). As a retrovirus, HIV-1 is characterized by high mutation and recombination rates, which are ascribed to the lack of proofreading activity and frequent template switching of its reverse transcriptase (RT) during viral DNA synthesis (Levy et al., 2004; Cuevas et al., 2015). The vast majority of HIV-1 infection worldwide were caused by HIV-1 M group, which is classified into 10 subtypes (A–D, F–L). Frequent HIV-1 coinfection and/or superinfection by different subtypes give the virus the chance to recombine and generate various inter-subtype recombinants. A circulating recombinant form (CRF) can be defined if it is identified from three or more epidemiologically unlinked individuals. When a recombinant virus is found only in one or two individuals, it is defined as a unique recombinant form (URF). Full-length genomic sequences are necessary for identifying and characterizing a nascent recombinant. Rapidly growing number of near full-length HIV-1 genomic sequences to be sequenced enables the finding and identification of new HIV-1 inter-subtype recombinants. So far, 140 CRFs have been identified and a large number of URFs were reported.^2^ It is foreseeable that a growing number of CRFs will be reported in the near future due to the high recombination potential of HIV-1. In this perspective paper, we aim to discuss the prevalence and clinical importance of various HIV-1 CRFs and propose how to report and make sense of a new HIV-1 CRF.

### 1.1 HIV-1 subtypes involved in recombinants

All known HIV-1 subtypes were involved in the formation of one or more CRFs (Table 1). As the most widely prevalent subtype, B subtype participated in more than half (57.1%) of all described CRFs, followed by subtypes C (22.1%), A (including various sub-subtypes) (21.4%), F (17.9%), and G (15.7%). On the other hand, the growing prevalence of some CRFs give them more chance to participate in the generation of second-generation recombinants, some of which were identified as new CRFs. For example, CRF01_AE, CRF02_AG, and CRF07_BC were involved in 40, 14.3, and 7.9% of all described CRFs, respectively.

**Table 1:** Contribution of HIV-1 subtypes to CRFs.

Subtypes/CRFs	Number	Percentage (%)
B	80	57.1
CRF01_AE	56	40.0
C	31	22.1
A	30	21.4
F	25	17.9
G	22	15.7
CRF07_BC	20	14.3
CRF02_AG	11	7.9
D	9	6.4
J	6	4.3
K	6	4.3
H	4	2.9
CRF06	3	2.1
CRF08	2	1.4
CRF55	2	1.4
CRF22_01A1	1	0.7
CRF27_cpx	1	0.7
CRF56_cpx	1	0.7
CRF94_cpx	1	0.7
CRF109_0107	1	0.7

### 1.2 Genomic sequences of HIV-1 CRFs

The development of next-generation sequencing (NGS) technologies and greatly reduced sequencing costs facilitate HIV-1 genome sequencing, which enables HIV-1 molecular epidemiological investigation and drug resistance monitoring. A large number of HIV-1 genomic sequences were reported and deposited in the LANL HIV database (Linchangco et al., 2021). According to the numbers of available partial and near full-length genomic sequences in the database, only two CRFs (CRF01_AE and CRF02_AG) had more than 100 available full-length genomic sequences, and the vast majority (85.9%) of CRFs had less than 10 full-length genomic sequences (Table 2). Compared to full-length genomic sequences, more partial genomic sequences of CRFs were sequenced. Twenty-seven (20%) CRFs had more than 100 available partial genomic sequences, and 57 (43.7%) CRFs had less than 10 available partial genomic sequences. Although more partial genomic sequences are available, some of them are short and contain few or no of critical recombination breakpoints to characterize a CRF. There are two reasons to explain why too few genomic sequences of some CRFs, especially the full-length genomic sequences, are available in the database. First, most CRFs had a very low prevalence, or only caused sporadic infections. Second, some CRFs might be prevalent, but were less frequently investigated due to low level of local socio-economic development. If this is true, the prevalence of some CRFs might have been underestimated.

**Table 2:** Available sequence information of 130 HIV-1 CRFs in the LANL HIV database.

Sequences number in the LANL HIV database	Partial sequences		Near full-length genomic sequences	
	CRFs number	Percentage (%)	CRFs number	Percentage (%)
>100	27	20	2	1.5
10–100	46	36.3	17	12.6
<10	57	43.7	116	85.9

A total of 135 CRFs that have information of sequence number were included in this table.

### 1.3 Prevalence of HIV-1 CRFs

Of 140 described CRFs, only few CRFs led to a widespread transmission and prevalence (Table 2; Tebit et al., 2007). CRF01_AE and CRF02_AG were the most widely circulating CRFs around the world, followed by CRF07_BC, CRF08_BC, CRF06_cpx, CRF55_01B, and CRF63_02A6. CRF01_AE, CRF02_AG, and CRF06_cpx showed a global prevalence and their genomic sequences had been reported in 19, 20, and 12 countries/regions, respectively (Supplementary Table S1). Some CRFs (e.g., CRF07_BC, CRF08_BC, CRF11_cpx, CRF35_A1D, CRF55_01B, and CRF63_02A6) had a regional prevalence. The prevalence of CRF07_BC, CRF08_BC, and CRF55_01B were mainly restricted to China and surrounding countries/regions, CRF63_02A6 was mainly circulating in Russia, and CRF35_A1D was mainly circulating in Middle-East countries (e.g., Iran and Afghanistan). For most CRFs, their earliest representative strains were from before 2020 (Supplementary Table S1), implying that they had enough time to spread and become epidemic. Few available sequences more likely reflect their very low prevalence.

At least 47 CRFs (33.6%) were identified in China (Supplementary Table S2). High frequency of CRFs occurring in China was mainly ascribed to co-circulation of several HIV-1 subtypes in high-risk groups such as injection drug users (IDUs) and men who have sex with men (MSM). For example, co-circulation of subtypes B and C among IDUs in early 1990s led to the generation of various CRFs_BC and a large number of URFs_BC (Yang et al., 2002; Pang et al., 2012; Li et al., 2016). Accompanied with rapidly growing prevalence of several CRFs, including CRF01_AE, CRF07_BC, CRF08_BC, and CRF55_01B among IDUs and/or MSM, the co-circulation of these CRFs with subtypes B and C led to the generation of some complex CRFs and URFs, including some second-generation recombinants. Of 47 CRFs identified in China, 22 (46.8%) belonged to second-generation and third-generation recombinants, including CRFs_0107, CRF_0108, CRF_0155, CRF_07B, CRF_0755, and CRF_07109 (Supplementary Table S3). The vast majority (90.9%) of these second-generation and third-generation recombinants were CRF07-related CRFs, including 17 CRFs_0107 that accounted for 77.3% of all these CRFs. CRFs_0107 has exceeded CRFs_BC (10, 45.5%) to be the most frequently occurring recombination forms in China, which was ascribed to the dominance of both CRF01_AE and CRF07_BC and their co-circulation among MSM and IDUs (Zhang et al., 2006; Zhao et al., 2016). Except CRF01_BC, CRF07_BC, CRF08_BC, and CRF55_01B, other CRFs and URFs originated in China did not cause local and national epidemic.

### 1.4 Phylogenetic analysis of partial *pol* sequences and the identification of new CRFs

HIV-1 *pol* gene encodes viral replication enzymes. Partial *pol* sequences covering protease (PR) and reverse transcriptase (RT) coding regions was generally required for HIV-1 genotypic drug resistance assays since most of currently used antiretroviral drugs target RT and PR (Zuo et al., 2020; Rhee et al., 2022). Drug resistance surveillance program facilitated the sequencing of PR-RT region of *pol* gene, and partial *pol* region had been the most sequenced genomic region of HIV-1 genome. Phylogenetic analyses of *pol* sequences can provide important insights into HIV-1 molecular epidemiology, drug resistance prevalence, as well as spatiotemporal spread (Wertheim et al., 2014; Rhee et al., 2022).

On the other hand, the *pol* gene is also a hot-spot region for HIV-1 recombination (Yang et al., 2002; Pang et al., 2012). Of 136 CRFs with available genome maps in the LANL HIV database, 106 (77.9%) CRFs had at least one recombination breakpoint in PR-RT region of *pol* gene. In the phylogenetic tree of *pol* sequences, the presence of one or more independent branches or clusters that do not closely cluster with any known HIV-1 subtypes and CRFs often imply new recombinant (CRF or URF) candidates to be identified by full-length genomic sequencing and recombination analyses (Topcu et al., 2022a). Transmission cluster analysis of *pol* sequences provides a robust way to find new CRFs (e.g., CRF87_cpx, CRF88_BC, and CRF91_cpx) from previously described URFs (Hu et al., 2017; Topcu et al., 2022a).

### 1.5 Origin, evolution, and clinical features of major CRFs

It is no doubt that the CRFs with high prevalence are of great clinical and epidemiological importance, and were largely concerned. The genomic sequence characteristics, origin, evolution, and expansion dynamics, as well as the clinical aspects of CRF01_AE, CRF02_AG, CRF07_BC, CRF08_BC, and CRF55_01B were previously well investigated. CRF01_AE is the earliest CRF to be identified. Its most recent common ancestor (MRCA) was estimated to emerge in Africa in the early 1970s, and spread to Asia in the early 1980s (Junqueira et al., 2020). CRF02_AG was demonstrated to originate in Central and West Africa in the late 1960s and spread to other continents since 1990s (Mir et al., 2016). Both CRF07_BC and CRF08_BC originated among IDUs in Yunnan, China in the early 1990s (Tee et al., 2008), and CRF55_01B among MSM in Shenzhen, China in about 2004 (Zhao et al., 2014).

Different CRFs appeared to be associated with different clinical features. Compared with B and CRF07_BC, CRF01_AE was associated with X4 tropism and rapid disease progression at least in Chinese patients (Li X. et al., 2014; Li Y. et al., 2014). In contrast, CRF07_BC was found to be more genetically conserved, and was associated with lower viral load and slower disease progression than other genotypes (Ye et al., 2022). CRF55_01B was associated with lower CD4+ T cell count and higher HIV RNA load than CRF01_AE in antiretroviral therapy (ART)-naïve individuals (Wei et al., 2021), which may contribute to its rapidly growing prevalence among MSM in recent year. For CRF02_AG, it appeared to have higher viral load than its parental strains (subtypes A and G), but did not show significant difference in clinical consequences from other genotypes (Fischetti et al., 2004; Konings et al., 2006; Njai et al., 2006).

## 2 Discussion

Host genetic and behavior factors, and genetic characteristics of the virus itself determine HIV-1 transmission (Carlson et al., 2014). However, the fact that only one or few CRFs stand out from many sister recombinants generated among the same high-risk groups during the same period and lead to rapid spread and expansion implies that host genetic and behavior factors might contribute relatively less to the expansion of a nascent CRF. A case in point is above-mentioned two closely genetically related CRFs 07_BC and 08_BC. Both CRFs originated among IDUs in southwestern Yunnan, China in the early 1990s, quickly spread to other regions and led to a national pandemic, whereas a large number (over 50% of all infections) of their sister URFs_BC (formed by recombination between subtypes B and C among IDUs), as well as other CRFs_BC (e.g., CRF62_BC, CRF64_BC, CRF88_BC, and CRF110_BC) from the same area during the same period did not lead to local or national epidemic (Yang et al., 2002; Pang et al., 2012).

Some studies demonstrated that sequence variations in viral protein and regulatory elements are the major determinants of HIV-1 transmission (Carlson et al., 2014; Ferdinandy et al., 2015; Tully et al., 2016). Although the molecular mechanism involving in the spread and epidemic of a nascent CRF remain largely unknown, the epidemiological success of a new recombinant or CRF is believed to be associated with increased viral transmissibility and/or fitness (such as enhanced *in vitro* replicative capacity and transmission potential, as well as the gaining of drug resistance and immune escape) since recombination provides an evolutionary shortcut to beneficial substitutions/mutations of their parental strains (Tebit et al., 2007; Ramirez et al., 2008; Tully et al., 2016; Cheng et al., 2022). The dominance of CRF02_AG was demonstrated to be associated with higher *in vitro* replicative capacity than its parental subtypes A and G (Konings et al., 2006; Njai et al., 2006). Similarly, we recently identified a new KE variant of CRF07_BC that carries five specific mutants in RT coding gene and shows higher *in vitro* replicative capacity than the wild type strain, and demonstrated that the new variant was responsible for the rapidly growing prevalence of CRF07_BC among MSM in recent year (Han et al., 2022). Almost all CRF55_01B were found to carry V179D/E, a polymorphic accessory NNRTI-selected mutation with low level resistance to efavirenz, which might be gained at its recombination origin between CRF01_AE and B (Liu et al., 2020; Zuo et al., 2020). Our recent study supported that all CRF55_01B carried V179D/E, and showed that CRF01_AE and CRF08_BC had similar drug residence prevalence, obviously higher than that of CRF07_BC, possibly suggesting different responses of different CRFs to ART (Zhang et al., 2024).

Although most described CRFs had very low prevalence and might be less clinical and epidemiological importance, the on-going generation of new HIV-1 URFs and CRFs still poses a major challenge for the prevention and control of HIV/AIDS. Therefore, there are two critical questions to be asked. Is a new CRF worth reporting by simply presenting its chimeric genomic map? and how to report and make sense of a new HIV-1 CRF?

Currently, most new CRFs were reported by simply providing the mosaic genomic maps, which are less valuable for the understanding of the origin, evolution, epidemiology, and biological and clinical characteristics of a new CRF. Additional analyses may be especially helpful and are recommended when reporting a new CRF. First, a comparative genomic analysis is recommended to reveal whether the new CRF carries specific mutations/substitutions in coding genes and regulatory elements relative to its parental strains or other genotypes (Ma et al., 2023). The variation information may provide potential candidates for molecular mechanism researches to determine the biological feature of the virus. Second, Bayesian phylogenetic analysis is suggested to infer the origin and population history of a new CRF (Suchard et al., 2018). Third, recent study showed that only near-full-length HIV-1 genomic sequences, rather than partial *pol* sequences, are sufficiently accurate for the determination of HIV-1 genotypic subtypes, especially CRFs (Topcu et al., 2022b). In spite of the advances in NGS, however, HIV-1 full-length genomic sequencing is still not practical for HIV-1 molecular epidemiological studies. Because the PR-RT region of *pol* region is frequently amplified and sequenced for drug resistance monitoring, it can provide a simple and affordable alternative to facilitate the molecular monitoring of newly identified CRFs. As mentioned above, however, at least 30 (22.1%) described CRFs do not contain any recombination breakpoints in PR-RT region. For the CRFs without recombination breakpoints in partial *pol* region, one and more additional representative genomic fragments that contain specific recombination breakpoints for distinguishing the CRF of interest from other CRFs are recommended. Forth, clinical and demographic information of the individuals carrying the CRF are helpful and can be provided and discussed. Furthermore, follow-up molecular epidemiological investigations focusing on high-risk cohorts for the new CRFs are especially encouraged.

In summary, the on-going generation of new complex HIV-1 URFs and CRFs brings a new challenge for the prevention and eradication of HIV/AIDS. Identification and prompt reporting of new CRFs will provide not only new insights into the understanding of genetic diversity and evolution of HIV-1, but also an early warning of potential prevalence of these variants. Apart from simply presenting the mosaic genomic maps, additional information, including the clinical and demographic data, genomic sequence characteristics, origin and evolutionary dynamics, as well as representative genomic fragments of the variants, are recommended to be provided when reporting new CRFs.

## Data availability statement

Publicly available datasets were analyzed in this study. This data can be found at: https://www.hiv.lanl.gov/components/sequence/HIV/crfdb/crfs.comp.

## Author contributions

ZW: Data curation, Formal analysis, Investigation, Methodology, Resources, Validation, Visualization, Writing – original draft. CZ: Conceptualization, Data curation, Formal analysis, Funding acquisition, Investigation, Methodology, Project administration, Resources, Supervision, Writing – original draft, Writing – review & editing.

## Footnotes

^1^https://www.unaids.org/en/resources/fact-sheet

^2^https://www.hiv.lanl.gov/components/sequence/HIV/crfdb/crfs.comp
