# Supplementary material for: How to report and make sense of a new HIV-1 circulating recombinant form?
Source: Front Microbiol. 2024 Feb 21;15:1343143. doi: 10.3389/fmicb.2024.1343143 (PMC10915052; doi:10.3389/fmicb.2024.1343143)
Supplement: Supplementary file 1 [file Data_Sheet_1.pdf]

Supplementary Material

Supplementary Methods:

To well understand the prevalence and clinical importance of HIV-1 CRFs, sequence information of all 140 described HIV-1 CRFs, including the numbers of partial and full-length genomic sequences, the sampling year and countries of full-length genomic sequences, parental subtypes/CRFs, and recombination breakpoints appearing in the PR-RT region of pol gene, were directly retrieved from the LANL HIV database (https://www.hiv.lanl.gov/content/sequence/HIV/mainpage.html), or obtained by re-counting and/or analyses according to data in the database. For example, we searched the full-length genomic sequences of each CRF using the Search Interface (https://www.hiv.lanl.gov/components/sequence/HIV/search/search.html), and then recorded all necessary information, including the number, sampling year and county of each full-length genomic sequence. Whether the PR-RT region of *pol* gene contains breakpoint point was determined based on the representative recombination map of each CRF that is available at https://www.hiv.lanl.gov/components/sequence/HIV/crfdb/crfs.comp.

Supplementary Table S1. Sequence information of CRFs with more than 100 partial or 10 full-length genomic sequences.

CRFs	Number of sequences in SeqDB	Near full-length genomic sequences				
		Number	Years	Number of Countries/regions	Countries/regions
CRF01_AE	81068	2126	1990-2019	19	Afghanistan, Australia, Belgium, Bulgaria, Cameroon, Central , African , Republic, China, Hong Kong, Indonesia, Iran, Japan, Laos, Philippines, Slovenia, Sweden, Thailand, United Kingdom, United States, Vietnam
CRF02_AG	28615	232	1991-2022	20	Angola, Belgium, Cameroon, Cote D'ivoire, Cyprus, France, Germany, Ghana, Guinea-Bissau, Liberia, Nigeria, Pakistan, Russian, Federation, Senegal, South Korea, Spain, Sweden, Uganda, United Kingdom,

					United States
CRF07_BC	21133	45	1997-2018	5	China, South Korea, Taiwan, United Kingdom, Vietnam
CRF08_BC	4446	37	1997-2007	1	China
CRF20_BG	266	27	1999-2019	3	Cuba, Spain, United States
CRF63_02A6	2254	26	2009-2019	1	Russian
CRF06_cpx	3364	25	1995-2021	12	Australia, Burkina Faso, Cameroon, China, Cyprus, Dem Rep Of Congo, Ghana, Mali, Nigeria, Russian Federation, Senegal, United Kingdom
CRF11_cpx	1473	25	1995-2016	6	Cameroon, Cyprus, France, Greece, Nigeria, Sweden
CRF35_A1D	1630	22	2005-2011	2	Iran, Afghanistan
CRF22_01A1	234	21	2001-2010	1	Cameroon
CRF140_0107	18	18	2020-2021	1	
CRF42_BF1	59	17	2003-2006	1	Luxembourg
CRF12_BF	526	14	1997-2018	4	Argentina, China, Spain, Uruguay
CRF14_BG	338	14	1999-2017	3	Cyprus, Portugal, Spain
CRF71_BF1	25	14	2002-2010	1	Brazil
CRF85_BC	253	11	2012-2014	1	China
CRF13_cpx	407	10	1996-2014	2	Cameroon, United Kingdom
CRF91_cpx	12	10	2017-2020	1	Cyprus
CRF103_01B	12	10	2015-2020	1	China
CRF55_01B	2435	9	2008-2015	1	China
CRF59_01B	348	9	2007-2012	1	China
CRF33_01B	246	8	2005-2007	2	Indonesia, Malaysia
CRF18_cpx	347	7	1997-2013	3	Cameroon, Cuba, United Kingdom
CRF25_cpx	154	6	2001-2006	3	Cameroon, Saudi Arabia, Dem Rep of Congo
CRF19_cpx	786	5	1999-2014	2	Cuba, Spain
CRF09_cpx	250	5	1995-2000	4	Cote D'ivoire, Ghana, Senegal, United States
CRF60_BC	192	5	2002-2011	3	France, Brazil, Italy
CRF45_cpx	159	5	1997-2004	4	France, Dem Rep of Congo, Cameroon, Gabon
CRF47_BF1	104	5	2008-2010	2	Spain, Brazil
CRF03_A6B	707	4	1997-2013	3	Belarus, Russian Federation, United Kingdom
CRF37_cpx	137	4	1997-2007	2	Cameroon, Cyprus
CRF10_CD	233	3	1996	1	Tanzania

Only CRFs with more than 100 partial or 10 full-length genomic sequences are shown in this table. The data was obtained from the LANL HIV database on Nov.17, 2023.

Supplementary Table S2. HIV-1 CRFs identified in China.

CRFs identified in China	Parental subtypes/CRFs
CRF07_BC	B, C
CRF08_BC	B, C
CRF55_01B	CRF01_AE, B
CRF57_BC	B, C
CRF59_01B	CRF01_AE, B
CRF61_BC	B, C
CRF62_BC	B, C
CRF64_BC	B, C
CRF65_cpx	CRF01_AE, B, C
CRF67_01B	CRF01_AE, B
CRF68_01B	CRF01_AE, B
CRF79_0107*	CRF01_AE, CRF07_BC
CRF80_0107*	CRF01_AE, CRF07_BC
CRF85_BC	B, C
CRF87_cpx	CRF01_AE, B, C
CRF88_BC	B, C
CRF96_cpx	CRF01_AE, B, C
CRF100_01C	CRF01_AE, C
CRF101_01B	CRF01_AE, B
CRF102_0107*	CRF01_AE, CRF07_BC
CRF103_01B	CRF01_AE, B
CRF104_0107*	CRF01_AE, CRF07_BC
CRF105_0108*	CRF01_AE, CRF08_BC
CRF106_cpx	CRF01_AE, B, C
CRF107_01B	CRF01_AE, B
CRF109_0107*	CRF01_AE, CRF07_BC
CRF110_BC	B, C
CRF111_01C	CRF01_AE, C
CRF112_01B	CRF01_AE, B
CRF113_0107*	CRF01_AE, CRF07_BC
CRF114_0155*	CRF01_AE, CRF55_01B
CRF115_01C	CRF01_AE, C
CRF117_0107*	CRF01_AE, CRF07_BC
CRF118_BC	B, C
CRF119_0107*	CRF01_AE, CRF07_BC
CRF120_0107*	CRF01_AE, CRF07_BC
CRF121_0107*	CRF01_AE, CRF07_BC
CRF123_0107*	CRF01_AE, CRF07_BC
CRF125_0107*	CRF01_AE, CRF07_BC
CRF126_0755*	CRF07_BC, CRF55_01B
CRF127_07109 #	CRF07_BC, CRF109_0107
CRF128_07B*	CRF07_BC, B
CRF134_0107*	CRF01_AE, CRF07_BC
CRF135_0107*	CRF01_AE, CRF07_BC
CRF136_0107*	CRF01_AE, CRF07_BC
CRF137_0107*	CRF01_AE, CRF07_BC
CRF140_0107*	CRF01_AE, CRF07_BC

* Second-generation CRFs; # Third-generation CRFs.

Because there was no pure E subtype to be found, CRF01_AE, the first defined CRF, was not considered as a real CRF. Therefore, CRF01_AE-involved CRFs were not considered as the second- and third-generation CRFs.

Supplementary Table S3. Second-generation recombinants occurring in China.

**Recombination forms**	**CRFs_0107**	**CRF_0108**	**CRF_0155**	**CRF_07B**	**CRF_0755**	**CRF_07109**	**Total**
Number	17	1	1	1	1	1	22
Percentage (%)	77.3	4.5	4.5	4.5	4.5	4.5	100

**Recombination forms**	**CRF07-involved CRFs**	**CRF08-involved CRFs**	**CRF55-involved CRFs**	**CRF109-involved CRFs**
Number	20	1	2	1
Percentage (%)	90.9	4.5	9.1	4.5
